# Severe acute respiratory syndrome (SARS) related coronavirus in bats

**DOI:** 10.1186/s44149-021-00004-w

**Published:** 2021-04-23

**Authors:** Rong Geng, Peng Zhou

**Affiliations:** 1 CAS key laboratory of special pathogens, Wuhan Institute of Virology, Chinese Academy of Sciences, Wuhan, China; 2 University of Chinese Academy of Sciences, Beijing, China

**Keywords:** SARS-related coronavirus, Bat, Cross-species, Spillover, Geographical distribution

## Abstract

Three major human coronavirus disease outbreaks, severe acute respiratory syndrome (SARS), Middle East respiratory syndrome (MERS) and 2019 coronavirus disease (COVID-19), occurred in the twenty-first century and were caused by different coronaviruses (CoVs). All these viruses are considered to have originated from bats and transmitted to humans through intermediate hosts. SARS-CoV-1 and SARS-CoV-2, disease agent of COVID-19, shared around 80% genomic similarity, and thus belong to SARS-related CoVs. As a natural reservoir of viruses, bats harbor numerous other SARS-related CoVs that could potentially infect humans around the world, causing SARS or COVID-19 like outbreaks in the future. In this review, we summarized the current knowledge of CoVs on geographical distribution, genetic diversity, cross-species transmission potential and possible pathogenesis in humans, aiming for a better understanding of bat SARS-related CoVs in the context of prevention and control.

## Introduction

Coronaviruses (CoVs) are a large family of positive-sense, single-strand RNA (+ssRNA) viruses that belong to the subfamily Orthocoronavirinae in the Coronaviridae family and the order *Nidovirales*. CoVs are enveloped viruses that have crown-like viral particles with the second largest genome among all RNA viruses, normally 27–32 kb in size.

CoVs were considered as causing only mild diseases in humans before 2002. The 2 human CoVs, HCoV-OC43 and HCoV-229E caused nonhospitalized respiratory infections, whereas the outbreak of SARS-CoV-1 in 2002 changed our view that CoVs can cause severe diseases in humans (Cui et al. 2019). The SARS-CoV-1 outbreak lasted for 9 months and resulted in 8098 people infected with 774 deaths, and caused the first global pandemic in the twenty-first century (Rota et al. 2003). Seventeen years later, another coronavirus related respiratory disease (COVID-19) outbreak (Huang et al. 2020; Zhou et al. 2020). The disease agent responsible for COVID-19 was identified as a novel SARS-related CoV, SARS-CoV-2 (Zhou et al. 2020). COVID-19 has spread to nearly all countries in the world and caused a serious public health crisis globally. To date (Aprial 24, 2021), there have been more than 100 million reported cases of infection and 2.5 million deaths (www.who.int/emergencies/diseases/novel-coronavirus-2019). SARS-CoV-2 shares 79.6% genome sequence identity and many biological features to SARS-CoV-1. Both viruses use the same cell entry receptor angiotensin-converting enzyme 2 (ACE2) for entry and cause severe pneumonia and systemic inflammatory diseases in humans (Hu et al. 2021; Zhou et al. 2020). However, there are also characteristics that are not shared by these 2 viruses. SARS-CoV-2 is more transmissible due to a high viral titer in the respiratory system early before the onset of symptoms, whereas high SARS-CoV-1 viral load can only be detected in more severe patients (Wölfel et al. 2020). This difference partialy explains why there are a lot more COVID-19 patients than SARS cases.

Bats are the second largest mammalian species in the world. They are also the only mammal with the capability of powered flight, which enables them to have a longer range of migration pattern when compared to land mammals. It is hypothesized that powered flight provides a selection pressure for bats that enabled them as ideal reservoir for viruses (Zhang et al. 2013). Correspondingly, bats are identified as natural reservoir hosts for many viruses, some of which were highly pathogenic for humans. For example, bat carried lyssaviruses (Rabies virus), henipaviruses (Nipah virus and Hendra virus) and filoviruses (Marburg virus and Ebola virus,) are all highly pathogenic to humans (Wang and Cowled 2015). In 2005, 2 independent groups reported the first finding of SARS-related CoVs (SARSr-CoVs) in Chinese horseshoe bats in China (Lau et al. 2005; Li et al. 2005). In the following years, more bat SARSr-CoVs were found worldwide, including the ones that are believed as ancestry viruses for SARS-CoV-1 and SARS-CoV-2 (Cui et al. 2019; Ge et al. 2012). In this review, we intend to summarize the current knowledge on genetic diversity and geographical distribution of bat SARSr-CoVs around the world, aiming for a better understanding of their cross-species transmission potential.

## Geographical distribution and diversity of bat SARSr-CoVs

SARSr-CoVs were first found in bats in molecular and serological surveillances of the natural reservoir of SARS-CoV-1 in 2005 (Lau et al. 2005; Li et al. 2005). Using pan-CoV detection primers targeting the viral RdRp gene, 2 groups reported bat CoV PCR-positive samples in multiple Rhinolophid bat species. They also found antibodies against SARS-CoV NP in 28–71% of bats. Subsequently, full-length genomes that shared 88–92% similarity to human SARS-CoV-1 were obtained (Lau et al. 2005; Li et al. 2005). These findings support the notion that bats are natural reservoir hosts of SARS-related CoVs.

**Fig. 1:**
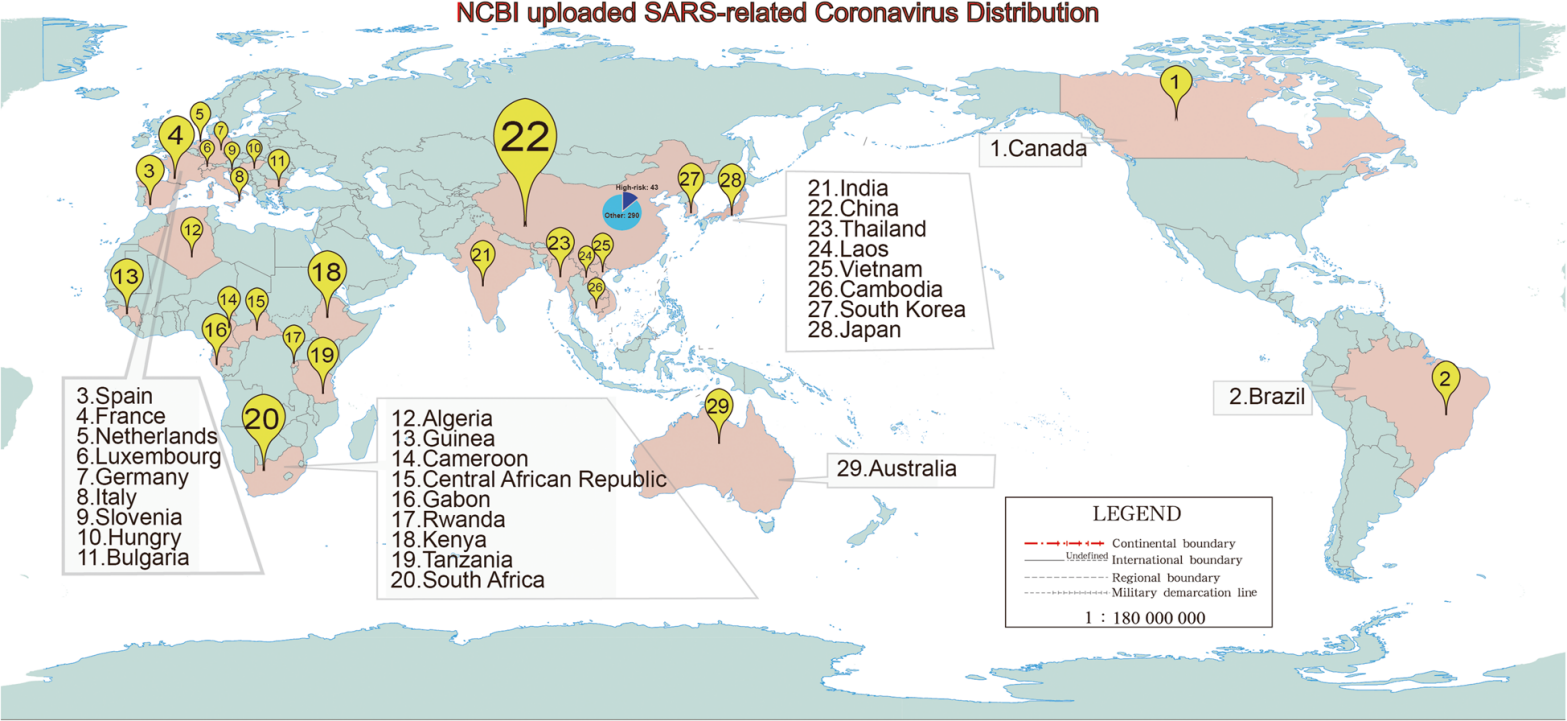
Geographical distribution of bat SARSr-CoVs (censor code of this map: S (2016) 1666). High-risk sequences, determined by a higher sequence similarity than WIV1 or RaTG13 to their respective reference sequences (SARS-CoV-1 or SARS-CoV-2). A conserved around 400 bp viral RdRp sequences were used for counting

The main bat species that carry SARSr-CoVs are families of *Rhinolophidae* and *Hipposideridae*, 2 insectivorous bats that are distributed widely in the world. Therefore, it’s expected that bat SARSr-CoVs should also be found in a wide range of countries (Fig. 1). So far, this viral family has been reported in China, South Korea, Japan, India, Burma, Thailand, Italy, Slovenia, Bulgaria, Kenya, Brazil and Australia that across 5 major continents in the world (Drexler et al. 2010; Lau et al. 2010; Lau et al. 2005; Lecis et al. 2019; Li et al. 2005; Rihtarič et al. 2010; Tong et al. 2009; Wacharapluesadee et al. 2015). Geographical distribution of SARSr-CoVs would be wider if more investigations are conducted in other countries. It’s also noticeable that the majority of viral sequences was found in China, where extensive investigations in looking for a possible animal origin of SARS-CoV-1 were conducted (Lau et al. 2005; Li et al. 2005).

**Fig. 2:**
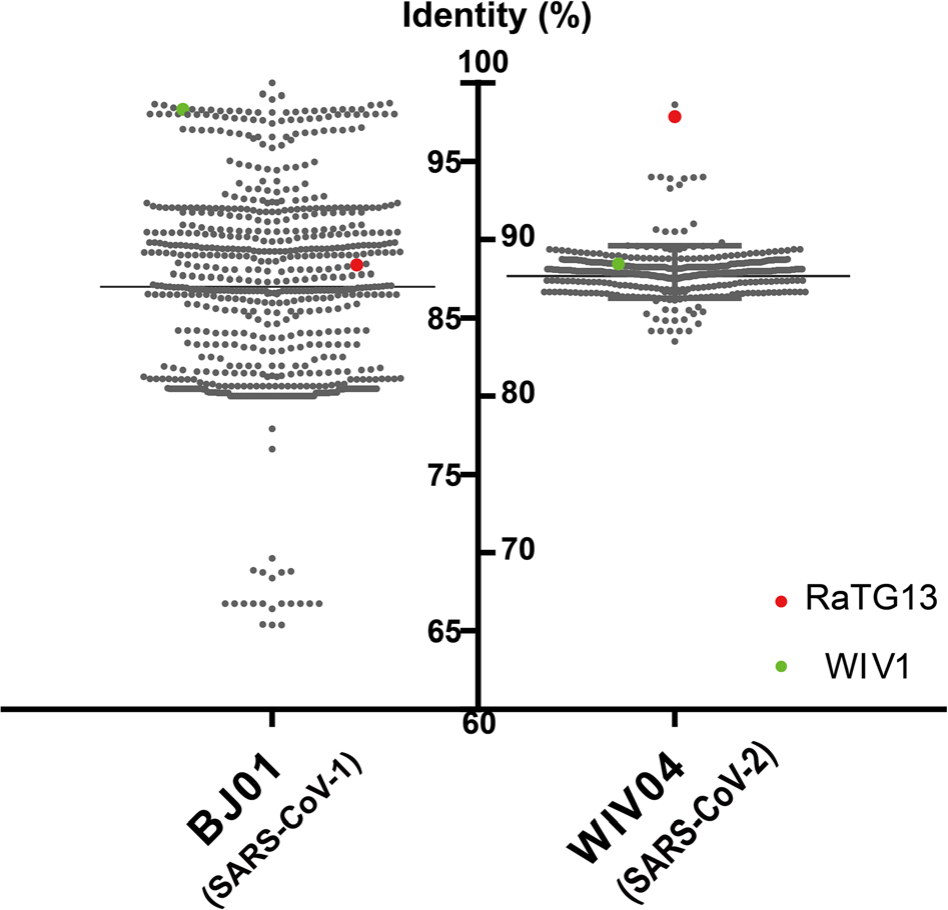
Genetic diversity of bat SARSr-CoVs. Partial RNA-dependent RNA polymerase (RdRp) sequences downloaded from NCBI database were aligned to the corresponding regions from SARS-CoV-1 (BJ01, accession number AY278488.2) or SARS-CoV-2 (WIV04, accession number MN996528.1). Their sequence identity to reference were showed. Bat SARSr-CoVs WIV1 (accession number KC881006.1) and RaTG13 (accession number MN996532.2) were highlighted in green or red

Genome changes caused by recombination, interchange and insertion or deletion are rather common among CoVs, particularly in bat CoVs (Su et al. 2016). As a consequence, 22 of the 39 ICTV CoVs species have been found in bats (Fan et al. 2019). It’s expected that bat SARSr-CoVs would show large genetic diversities. Due to the large genome size and high genetic diversity, full-length genome of bat CoVs was not readily obtainable, particularly before the application of next generation sequencing (NGS) technology. Most of bat CoV surveillances were based on PCR targeting at around 400 bp partial RdRp fragment, which is the most conserved region among CoVs (Lau et al. 2010). So far, there are more than 300 SARSr-CoVs partial RdRp sequences in the NCBI database. Identities of these sequences range from 85 to 99% to human SARS-CoV-1 or 70–99% to SARS-CoV-2 reference sequences (Fig. 2). The high degree of genetic diversity was probably shaped by high host species diversity, viral recombination caused by frequent cross-species transmission within *Rhinolophus* bats, and independent evolution due to a wide geographical distribution (Fan et al. 2019; Latinne et al. 2020). In general, there are more than 70 sequences that shared higher than 95% identity to SARS-CoV-1, whereas there are only 2 closely related to SARS-CoV-2, suggesting bats have a higher chance to carry SARS-CoV-1 and its close relatives. Notably, this bias might be affected by sampling timing, location or bat species during investigation. Since most of bat SARSr-CoV sequences were from Chinese bats, it is possible that more SARS-CoV-2 related sequences could be found in bats elsewhere.

## Potentials for cross-species transmission of bat SARS-related CoVs

**Fig. 3:**
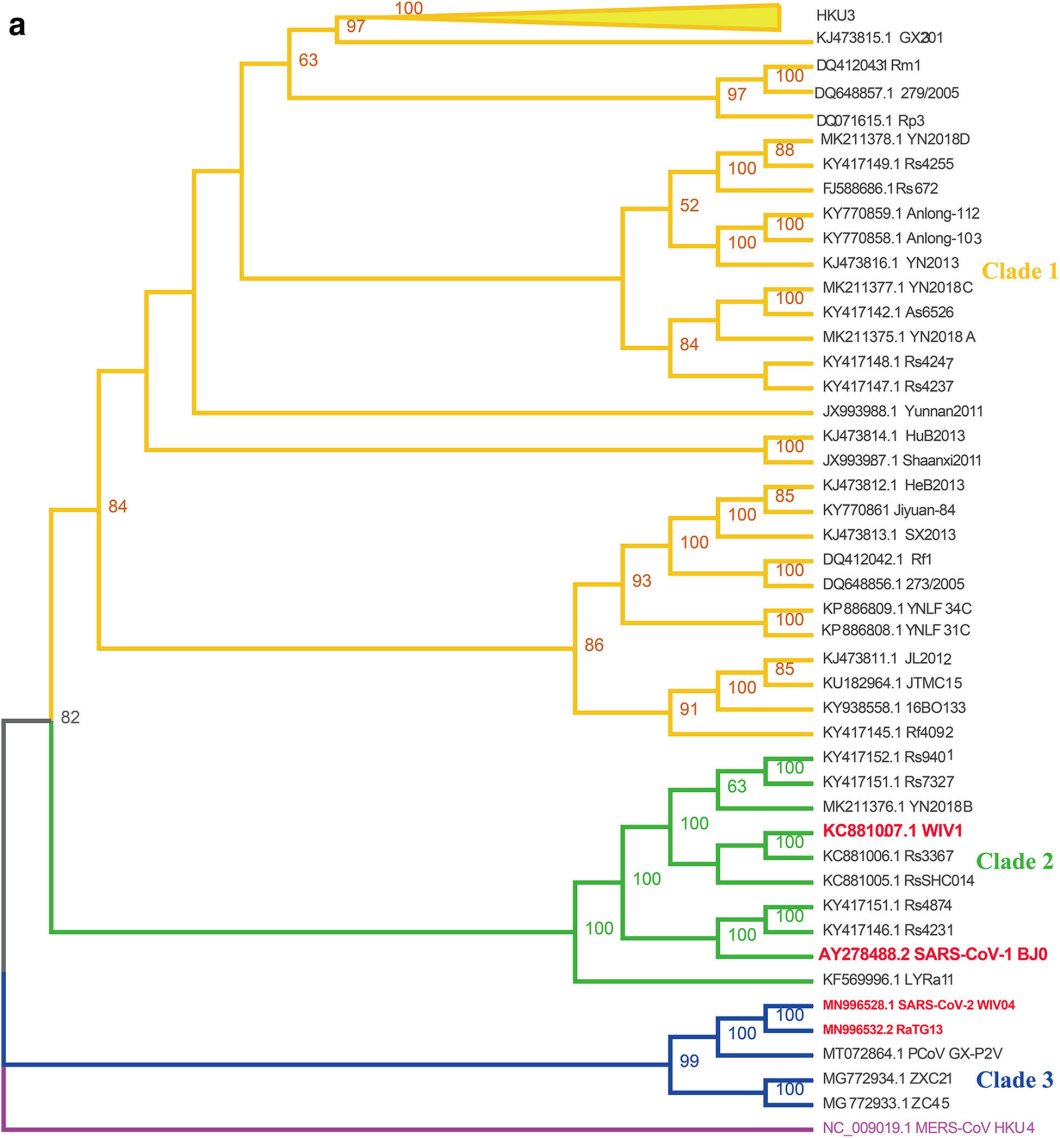

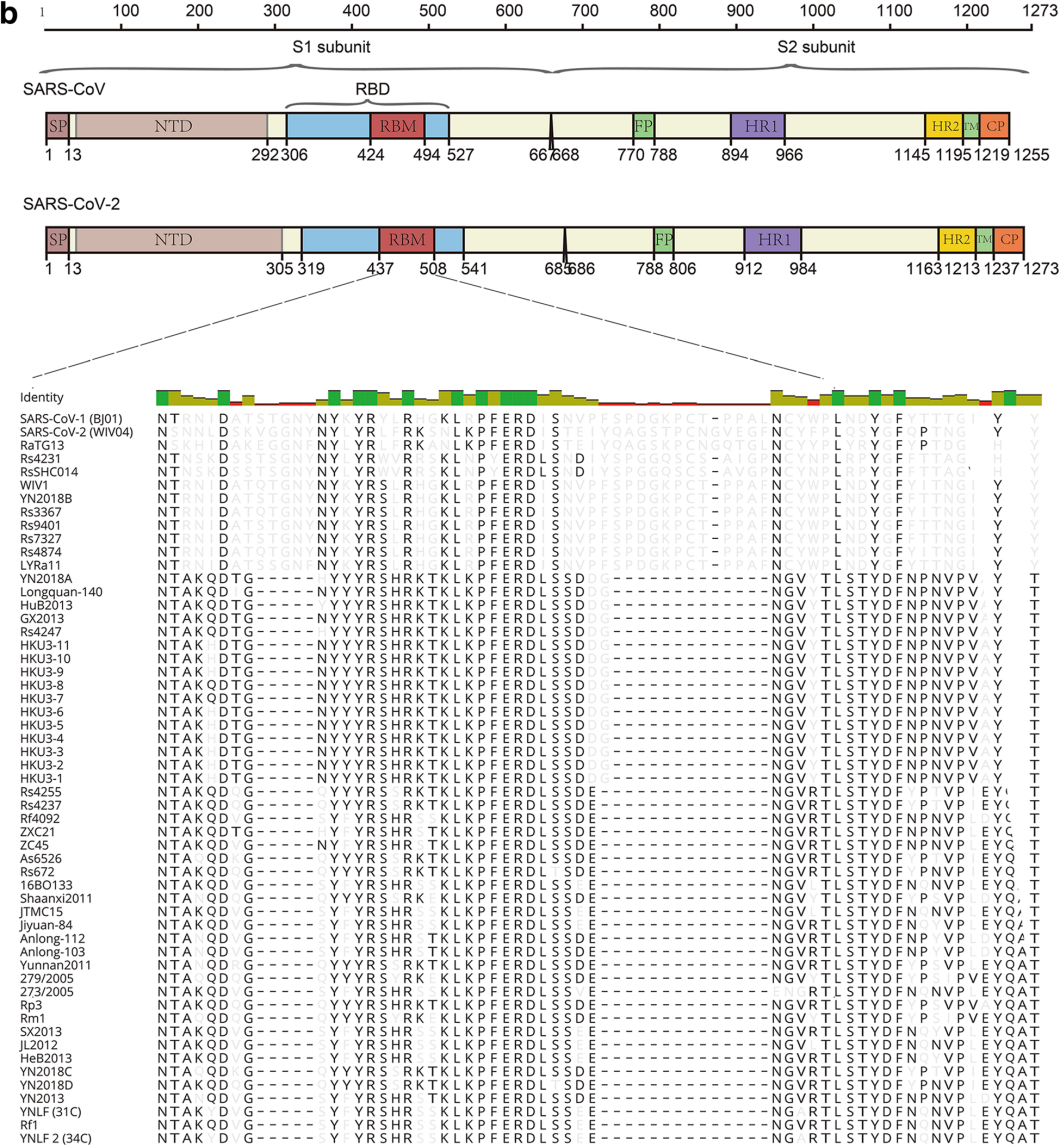
Spike gene analysis of bat SARSr-CoVs. **a** Phylogenetic tree of bat SARSr-CoVs S full-length sequences from NCBI database. Three different clades were shown. Bat MERS-related HKU4-CoV was used as outgroup. **b** Alignment of bat SARSr-CoVs receptor-binding motif (RBM) in spike protein. The residue numbers refer to corresponding regions in SARS-CoV-1 or SARS-CoV-2. Please refer to (**a**) for accession number or clade information for each viral strain. Dashes indicated residues absent. SP, signal peptide; NTD, N-terminal domain; FP, fusion peptide; HR1, heptad repeat 1; HR2, heptad repeat 2; TM, transmembrane domain; CP, cytoplasmic domain

Coronavirus spike-receptor binding has been believed to be the most important constraint to infection in a new host population, and albeit protease cleavage may represent another critical barrier to zoonotic CoV infection (Menachery et al. 2020). Based on phylogenetic analysis of spike genes, the 60 available SARSr-CoVs sequences can be grouped into 3 different clades: SARS-CoV-2 and its close relatives (clade 3), SARS-CoV-1 and its close relatives (clade 2), and those distant from both human viruses (clade 1) (Fig. 3). Both SARS-CoV-1 and SARS-CoV-2 utilized human ACE2 as cell entry receptor. It’s conceivable that those bat SARSr-CoVs utilize human ACE2 (such as WIV1-CoV) pose higher cross-species risk than viruses that don’t use ACE2 for entry (such as Rp3-CoV and Rm1-CoV) (Ge et al. 2013; Zhou et al. 2020). Notably, these high-risk viruses were only found in Yunnan Province in China so far. Whether or not these viruses distribute in other provinces or countries is yet to be investigated. Moreover, frequent monitoring clade 2 and clade 3 viruses in certain bat colonies should also be conducted in the future.

Coronavirus cell entry is mainly mediated *via* spike protein (S), which binds to ACE2 receptor through receptor-binding motif (RBM). During SARS-CoV-1 infection, S1-S2 is cleaved by protease in the endosome followed by viral-cell membrane fusion. Subsequently, the viral RNA is injected into the cytoplasm (Hu et al. 2021). In SARS-CoV-2, an insertion of 4 amino acid (PRRA) during junction of S1 and S2 enables more effective cleavage by furin protease than SARS-CoV-1 (Hu et al. 2021). Due to the important role of CoV S protein in viral entry and transmission, any mutation could potentially affect biological characteritics of SARSr-CoV. For example, a single D614G residue mutation greatly increased the transmissibility of SARS-CoV-2 among humans (Hou et al. 2020). Likewise, mink SARS-CoV-2 cluster 5 viruses have lowered immune reactivity against convalescent serum from human COVID-19 patients, probably due to several mutations in RBM of viral S protein (European Centre for Disease Prevention and Control 2020; Wölfel et al. 2020).

Based on RBM differences, bat SARSr-CoV can be divided into 2 clusters. In most of bat SARSr-CoVs (cluster 1), there are two 5 or 14 amino acid gaps that disabled ACE2 binding ability, and were subsequently considered as low risk viruses (Hu et al. 2021). In contrast, a small cluster of bat SARSr-CoVs possesses similar RBM as human SARS-CoV-1/2 viruses (cluster 2), including WIV1-CoV and RaTG13-CoV. These viruses have the potential to cause spillover events (Hu et al. 2021). Notably, all cluster 2 viruses were found in Yunnan Province in China, indicating the ancestral virus for SARS-CoV-1/2 was possibly from this area. However, it should not be ruled out the possibility that SARS-CoV-1/2 is originated outside China since samples have so far only been collected from Yunnan and Southeast Asian countries bordered with Yunnan. Recently, 2 SARS-CoV-2 related viruses have been discovered in *Rhinolophus* bats in Japan and Cambodia (Mallapaty 2020). The potential of cross-species transmission of bat SARSr-CoV has been demonstrated by the fact that antibodies against SARSr-CoV were found in humans who has no prior exposure history to SARS-CoV in Yunnan (Wang et al. 2018).

## Potential pathogenicity of bat SARS-related CoVs to humans and other animals

There are many researches about organ tropism of virus infection in COVID-19 patients (Deshmukh et al. 2021; Jiang et al. 2020). Both SARS-CoV-1 and SARS-CoV-2 are highly transmissible and cause severe diseases or even death in humans (Cui et al. 2019; Hu et al. 2021). Animal models used for assessment of viral pathogenicity have been tested for these 2 viruses, including non-human primates (rhesus macaques, cynomolgus monkeys, marmosets and African green monkeys), transgenic mice carrying human ACE2 or wild-type mice (using mouse-adapted virus), ferrets, gold hamsters and minks (Hu et al. 2021; Jiang et al. 2020; Munster et al. 2020; Oreshkova et al. 2020; Shi et al. 2020). In these models, SARS-CoV-2 caused mild to severe diseases similar to human infections depends on the models. In human ACE2-transgenic mice, SARS-CoV-2 infection localized to lungs and resulted in severe interstitial pneumonia, but the viral tissue tropism was somewhat different from humans. While it only induced mild diseases in monkeys (Munster et al. 2020). Moreover, other animals have also been used for SARS-CoV-2 infection, studies demonstrated that it could infect hamsters, ferrets and cats with resulting mild symptoms but not pigs, chickens or ducks (Oreshkova et al. 2020; Shi et al. 2020).

In contrast to a frequent transmission of CoVs from bats to humans, pigs and other mammals, little is known about the potential pathogenicity of bat SARSr-CoVs in other spillover hosts (such as caw and horse). A recombinant bat HKU3-CoV with RBM replaced from human SARS-CoV-1 was found to replicate well in human airway epithelial cells, suggesting that bat HKU3-CoV has the potential to cause diseases in humans once it gains the ability to infect (Becker et al. 2008). In another study, SARS-CoV-1 baring a bat SARSr-CoV SHC014 spike was found to efficiently use ACE2 replicate in primary human airway cells, and achieve *in vitro* titers equivalent to epidemic strains of SARS-CoV-1 (Menachery et al. 2015). Full-length SHC014 recombinant virus also demonstrated robust viral replication in human ACE2 transgenic mouse. This virus could not be neutralized by human SARS-CoV-1 antibodies, indicating the potential threat to humans once the species barriers are crossed (Menachery et al. 2015). On the other hand, another bat SARSr-CoV WIV1-CoV may pose little threat to humans since it caused only mild diseases in human ACE2 transgenic mice and it can be neutralized effectively by human SARS-CoV-1 antibodies (Menachery et al. 2016). Taken together, these studies indicated that further adaptation to an intermediate host such as palm civets or pangolines may be needed for a bat SARSr-CoV to jump over to humans (Kan et al. 2005; Xiao et al. 2020).

## Special bat immune system

Although bat SARSr-CoVs can cause diseases in mice, these viruses are carried by bats for long-term without causing any diseases (Wang and Cowled 2015). Actually, bats are found as natural reservoirs for CoVs, and the average positive rate of CoVs in bat population is around 10% (Lau et al. 2005; Li et al. 2005; Tang et al. 2006). Moreover, bats experimentally infected with bat SARSr-CoV WIV1 or other viruses such as Hendra virus survived with little tissue damage (van Doremalen et al. 2018; Williamson et al. 2000). It has been known for some time that bats may have unique antiviral defense system that entitled themselves quickly inhibit viral infection. In recent years, there have been accumulating evidences pointing to a unique antiviral system in bats. It has been found that Pteropid bats maintain a much-contracted type I interferon locus, but its interferon α keeps a constitutive expression pattern which cannot be downregulated by viral infection (Zhou et al. 2016). This unique expression pattern may allow bats quickly summon up antiviral innate immune responses.

On the other hand, overreaction is one of the pathogenic mechanisms by which virus infection causes human disease. For example, severe cytokine storm triggered by SARS-CoV-2 infection eventually caused multi-organ damages in humans (Costela-Ruiz et al. 2020). In contrast, bats have evolved to a dampened inflammatory response. Point mutations found in STING and NLRP3 proteins, key mediators of viral induced interferon production pathway or inflammation pathways respectively, caused dampened interferon and inflammatory responses following infection (Ahn et al. 2019; Xie et al. 2018). It has been hypothesized that these genetic differences contribute to a special relationship between bats and viruses.

## Future perspectives

It has long been believed that outbreaks of emerging virus diseases in humans will most likely be from wildlife animals. SARS-CoV is among the list of prioritizing viruses for research and development in emergency contexts by the World Health Organization since bat SARSr-CoVs could cause another SARS-like pandemic (Mehand et al. 2018). The devastating outcomes caused by SARS-CoV-2 to human society further demand a better understanding of this group of viruses. It is impossible to predict exactly which bat virus would cause the next outbreak, however, active surveillance of SARSr-CoVs in bat population and constant monitoring of the genetic changes that may allow which infect humans may help us to identify the hotspot areas of spillover events that might occur. On the other hand, banning wildlife markets would minimize the chance of bat SARSr-CoVs spillover to humans *via* an intermediate host. Banning bat hunting and keeping humans off to the bat natural environment would also decrease chance of future viral spillover. Several SARS-CoV-2 vaccines have been licensed for emergency use worldwide, for example mRNA-1273 (Moderna), BBIBP-CorV (Sinopharm) (Krammer 2020), that will facilitate the control of the current COVID-19 pandemic.
